# Quercetin ameliorates Aβ toxicity in *Drosophila* AD model by modulating cell cycle-related protein expression

**DOI:** 10.18632/oncotarget.11963

**Published:** 2016-09-10

**Authors:** Yan Kong, Ke Li, Tingting Fu, Chao Wan, Dongdong Zhang, Hang Song, Yao Zhang, Na Liu, Zhenji Gan, Liudi Yuan

**Affiliations:** ^1^ Department of Biochemistry and Molecular Biology, Medical School, Southeast University, Nanjing, Jiangsu, China; ^2^ Gladstone Institute of Cardiovascular Disease and Department of Pharmaceutical Chemistry, University of California, San Francisco, CA, USA; ^3^ MOE Key Laboratory of Model Animal for Disease Study, Model Animal Research Center, Nanjing University, Nanjing, China; ^4^ State Education Ministry's Key Laboratory of Developmental Genes and Human Diseases, Institute of Life Sciences, Southeast University, China

**Keywords:** quercetin, Drosophila, Alzheimer's disease, cell cycle, DNA replication, Gerotarget

## Abstract

Alzheimer's disease (AD) is a prevalent neurodegenerative disorder characterized by β amyloid (Aβ) deposition and neurofibril tangles. It has been reported that a bioflavonoid, quercetin, could ameliorate AD phenotypes in *C. elegans* and mice. However, the mechanism underlying the ameliorative effect of quercetin is not fully understood yet. *Drosophila* models could recapitulate AD-like phenotypes, such as shortened lifespan, impaired locomotive ability as well as defects in learning and memory. So in this study, we investigated the effects of quercetin on AD in *Drosophila* model and explored the underlying mechanisms. We found quercetin could effectively intervene in AD pathogenesis *in vivo*. Mechanism study showed quercetin could restore the expression of genes perturbed by Aβ accumulation, such as those involved in cell cycle and DNA replication. Cyclin B, an important cell cycle protein, was chosen to test whether it participated in the AD ameliorative effects of quercetin. We found that cyclin B RNAi in the brain could alleviate AD phenotypes. Taken together, the current study suggested that the neuroprotective effects of quercetin were mediated at least partially by targeting cell cycle-related proteins.

## INTRODUCTION

Alzheimer's disease is a prevalent neurodegenerative disorder that mainly affects the elderly population. Its pathological features include senile plaques formed by Aβ deposition and neurofibril tangles composed of hyper-phosphorylated microtube associated tau protein [1]. Aβ is derived from amyloid protein precursor (APP) which should be sequential processed by β secretase (BACE1) and γ secretase [2]. γ secretase is a protease complex formed by presenilin 1/2 (PS1/2), nicastrin, anterior pharynx-defective 1 (APH-1) and presenilin enhancer 2 (PEN-2) [3]. Mutations of APP and/or PS1/2 are usually found in early onset familial AD. However, the majority of AD cases are sporadic and late-onset. Its etiology is still elusive. Further investigation is required to elucidate detailed mechanisms and develop effective therapeutic methods. With short lifespan and convenience for genetic manipulation, *Drosophila* is extensively used in research for aging and aging related neurodegeneration [4, 5]. *Drosophila* AD models have been established by expressing human wildtype or Arctic mutant Aβ_42_ in central nervous systems [6, 7]. These flies demonstrate shorter lifespan, impaired locomotive ability and defects in learning and memory [6]. *Drosophila* models are widely used in exploring molecular mechanisms for AD pathogenesis and screening for anti-Alzheimer drugs [8].

Natural and chemically synthesized small molecules targeting important signaling pathways are widely used in research and clinic [9, 10]. Quercetin is a flavonoid enriched in plants such as onions, apples and tea. With the effects of antioxidant, radical-scavenging, anti-inflammation and anti-proliferation, quercetin has been reported to have the potential for treatments of cancer, cardiovascular disease, diabetes, infection, inflammation and neurodegeneration [11-15]. Quercetin protects primary neurons and hippocampal cultures against Aβ_42_ toxicity *in vitro* [16, 17]. In addition, quercetin could also ameliorate AD phenotypes *in vivo*. It could activate protein degradation pathways and protect *C. elegans* from Aβ_42_ induced paralysis [18]. In aged triple transgenic AD mice (3xTg-AD), quercetin decreases extracellular β-amyloidosis, tauopathy, astrogliosis and microgliosis in the hippocampus and the amygdala [19]. However, the detailed mechanism underlying the ameliorative effect of quercetin on AD is not fully understood yet.

Quercetin used in our study was purified from the flowers of *Styphnolobium japonicum*, one of the 50 fundamental herbs used in traditional Chinese medicine [20]. Using *Drosophila* AD models, we found that quercetin could extend the lifespan and rescue locomotive defects of AD flies. Transcriptomic analysis showed that several signaling pathways such as cell cycle proteins in FoxO signaling pathway and DNA replication were dysregulated in AD flies. Interestingly, dietary quercetin supplementation could restore Aβ induced perturbation on these pathways. Further experiments using *in vivo* RNAi of the cell cycle protein cyclin B in the brain ameliorated AD phenotypes, which confirmed that the beneficial effects of quercetin in AD was mediated by targeting cell cycle related proteins. In conclusion, our study validated the theory that ectopic cell cycle events could mediate neurodegeneration and confirmed that neurons exhibited biomarkers of cell cycle progression and DNA replication in AD brains using *Drosophila* model. Moreover, we revealed quercetin as a potential chemical to rescue AD phenotypes by acting on the expression of genes related to cell cycle events.

## RESULTS

### Quercetin rescued AD phenotypes in *Drosophila* model

**Figure 1:**
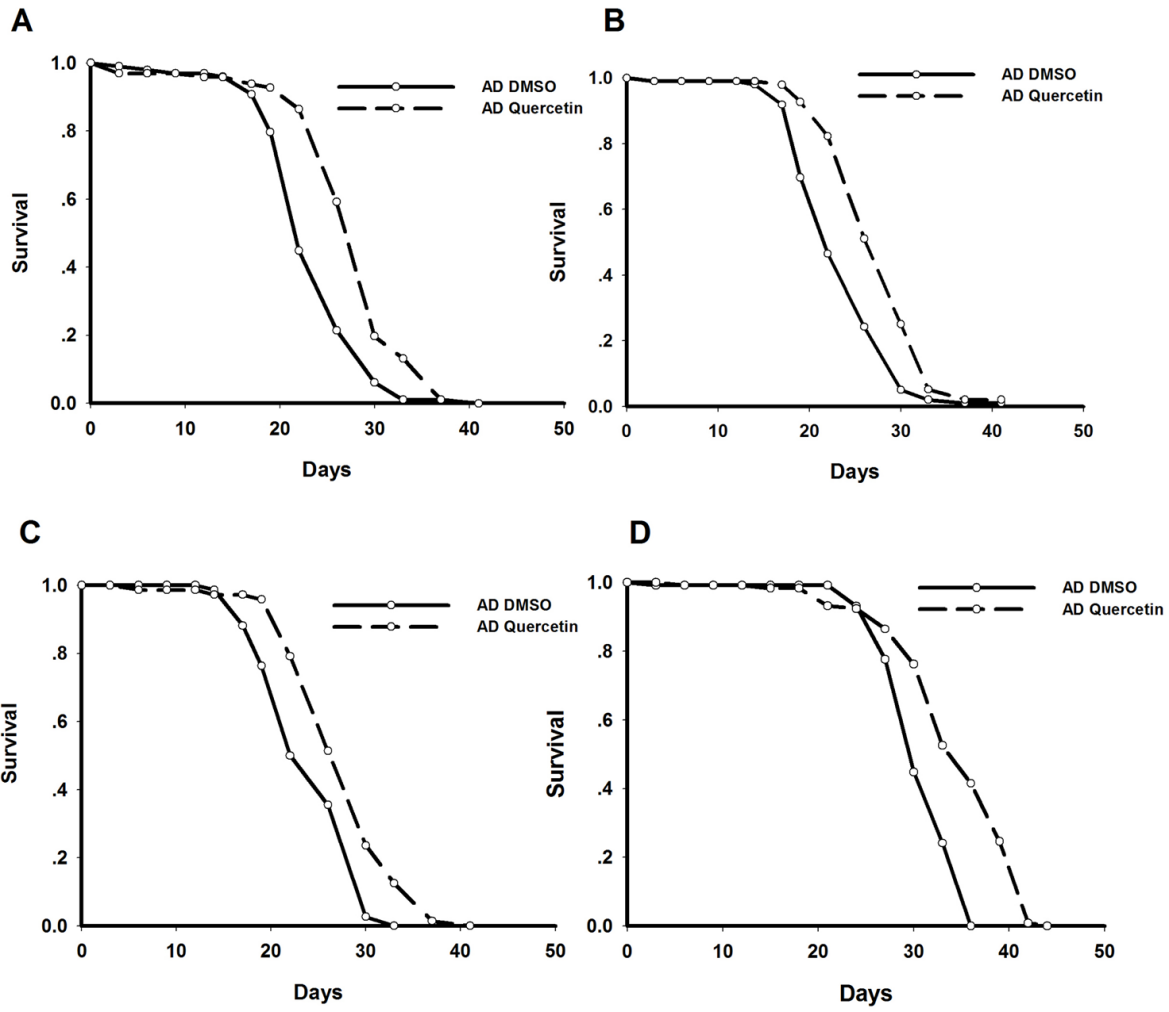
Quercetin extended lifespan of *AD Drosophila* Representative results from 4 independent experiments were shown. Survival curves were compared using the log-rank test (*P* < 0.05 between AD DMSO and AD Quercetin flies).

**Figure 2:**
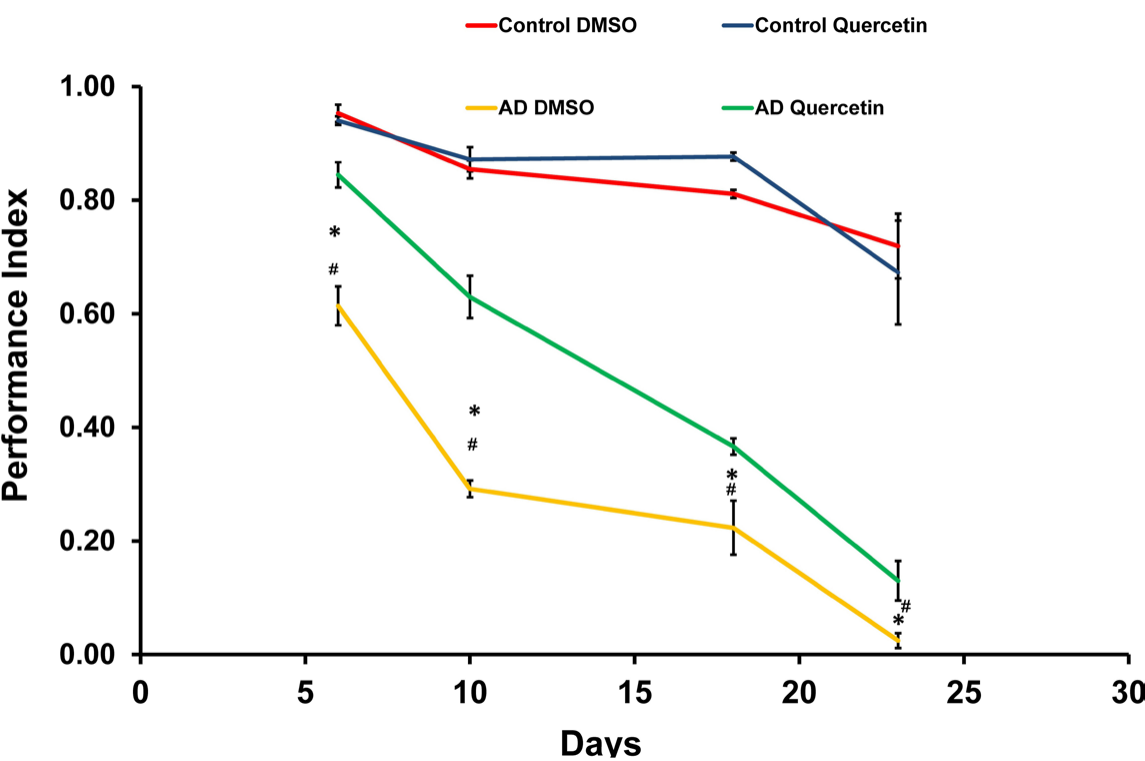
Quercetin ameliorated impaired climbing ability of *AD Drosophila* Climbing abilities were presented as the average performance index (PI) ±SEM (* *P* < 0.05 between Control DMSO and AD DMSO flies. # *P* < 0.05 between AD DMSO and AD Quercetin flies).

As reported previously, we established *Drosophila* AD model by driving Arctic Aβ_42_ expression in the brain [6, 7]. Specifically, *elav*-Gal4 virgins were crossed with UAS-Arctic Aβ_42_ males to induce pan-neuronal transgene expression in the offspring. We found AD flies recapitulated shorter lifespan and impaired locomotive behavior. Quercetin used in this study was extracted from *Styphnolobium japonicum* flowers. HPLC analysis showed 97.686% of the extract was quercetin dehydrate (Figure S1). We also performed mass spectrometry and ^13^C NMR to analyze the extract. The results of molecular weight and chemical structure further verified quercetin we used (Figure S2 and S3). Dietary supplementation of quercetin (0.44g/L in standard sugar-yeast medium) from adulthood dramatically and consistently extended lifespan of AD *Drosophila* in independent experiments (Figure 1). In addition, it could also rescue impairments in climbing ability (Figure 2). Taken together, our results indicated that quercetin could ameliorate Aβ toxicity *in vivo*.

### Aβ neurotoxic effects were mediated by cell cycle related signaling pathways *in vivo*

**Figure 3:**
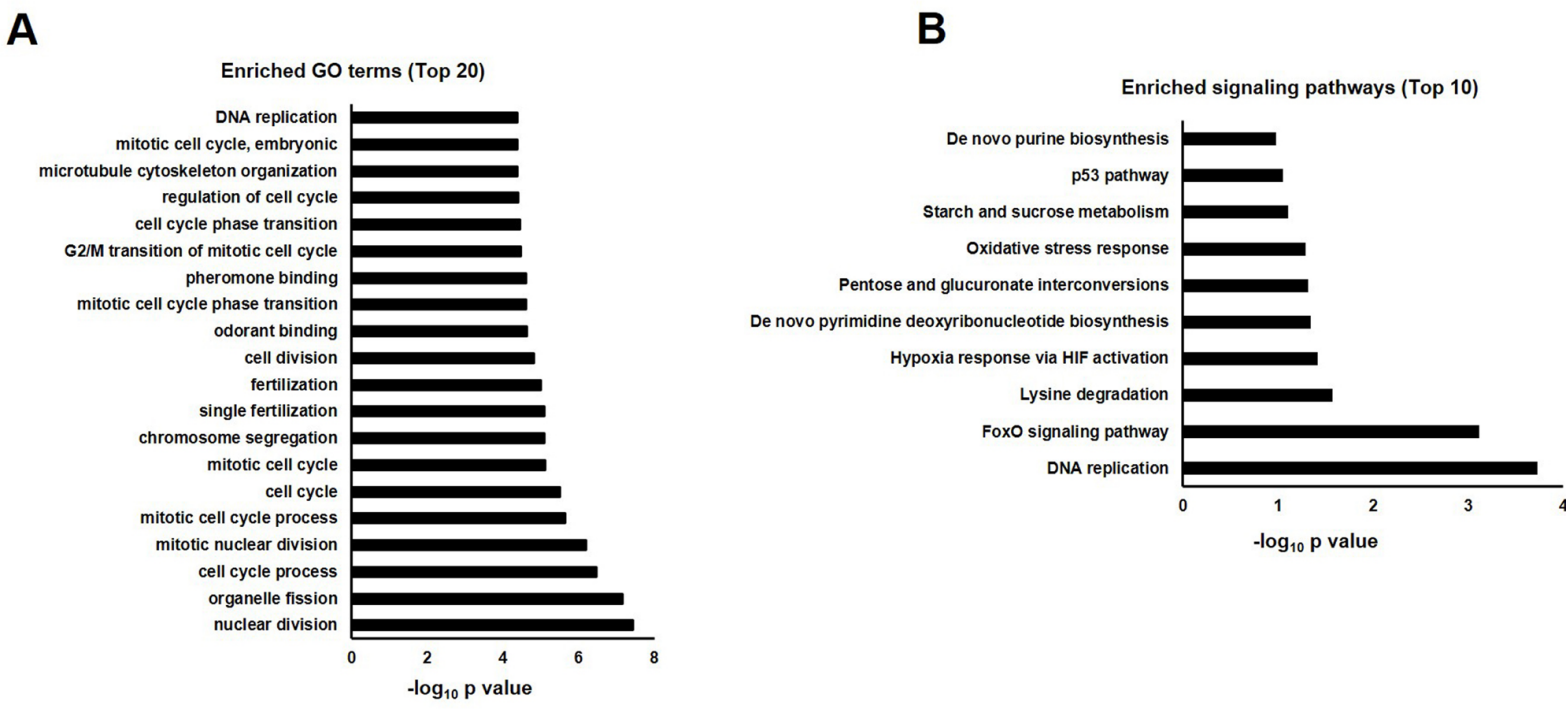
GO annotation and pathway enrichment of dysregulated genes in AD flies as compared with WT (AD *vs*. WT). **A.** Functional annotations were performed to analyze dysregulated genes in biological process, molecular function and cell component in AD *Drosophila*. Top 20 GO terms were shown and displayed as -log_10_ *P* values. **B.** Dysregulated genes were analyzed by KEGG pathway, PID Curated, PID BioCarta, PID Reactome, BioCyc, Reactome and Panther. Top 10 enriched pathways were shown and displayed as -log_10_ *P* values.

In order to investigate the underlying mechanisms for quercetin neuroprotective effects against Aβ, transcriptomic analysis was performed by Affymetrix *Drosophila* Genome 2.0 Array (Figure S4). At day 10 post eclosion, AD flies showed impaired climbing ability while their survival was unchanged. We chose female flies at this time point for transcriptomic analysis. Robust multi-array average (RMA) method was used to identify differentially expressed genes (ratio≥2 or ≤0.5). We found 47 transcripts were downregulated while 105 transcripts were increased in AD flies when compared with WT (*elav > w1118*) group. Gene ontology (GO) analysis was performed to classify dysregulated genes into different functional categories. Top 20 clusters were shown in Figure 3A.

Pathway enrichment analysis showed that DNA replication and cell cycle proteins in FoxO signaling pathway were significantly influenced by Aβ expression (Figure 3B). These results were highly consistent with GO analysis which showed cell cycle and DNA replication were perturbed in AD flies. Dysregulated genes involved in these pathways were listed in Table 1.

**Table 1:** Dysregulated pathways in AD flies as compared with WT *Drosophila*

Pathway	genes	up or down regulated
DNA replication	Mcm7, Mcm3, PCNA, Mcm2	up
FoxO signaling pathway	CycB, CG10924, polo, CycB3	up
Lysine degradation	Su, CG10814	down
Hypoxia response via HIF activation	dhd	up
*De novo* pyrimidine deoxyribonucleotide biosynthesis	RnrS	up
Pentose and glucuronate interconversions	UGP, Ugt86Dd	down
Oxidative stress response	dhd	up
Starch and sucrose metabolism	UGP, Ugt86Dd	down
p53 pathway	CycB	up
*De novo* purine biosynthesis	RnrS	up
Nitrogen metabolism	CG18673	up
Base excision repair	PCNA	up
Mismatch repair	PCNA	up
FGF signaling pathway	a5	down
EGF receptor signaling pathway	a5	down
Dorso-ventral axis formation	orb	up
Glycine, serine and threonine metabolism	Gld	up
Ascorbate and aldarate metabolism	Ugt86Dd	down
Retinol metabolism	Ugt86Dd	down
Galactose metabolism	UGP	down
Nucleotide excision repair	PCNA	up
Porphyrin and chlorophyll metabolism	Ugt86Dd	down
Citrate cycle (TCA cycle)	CG10924	up
Amino sugar and nucleotide sugar metabolism	UGP	down
Pyruvate metabolism	CG10924	up
Drug metabolism - other enzymes	Ugt86Dd	down
Glycolysis /Gluconeogenesis	CG10924	up
Drug metabolism -cytochrome P450	Ugt86Dd	down
Metabolism of xenobiotics by cytochrome P450	Ugt86Dd	down
Glutathione metabolism	RnrS	up
Phagosome	alphaTub67C	up
Pyrimidine metabolism	RnrS	up
Ubiquitin mediated proteolysis	APC7	up
Purine metabolism	RnrS	up
Metabolic pathways	Gld, RnrS, CG10924	up
	Ugt86Dd,UGP	down

### Quercetin could restore signaling pathways interrupted by Aβ expression

**Figure 4:**
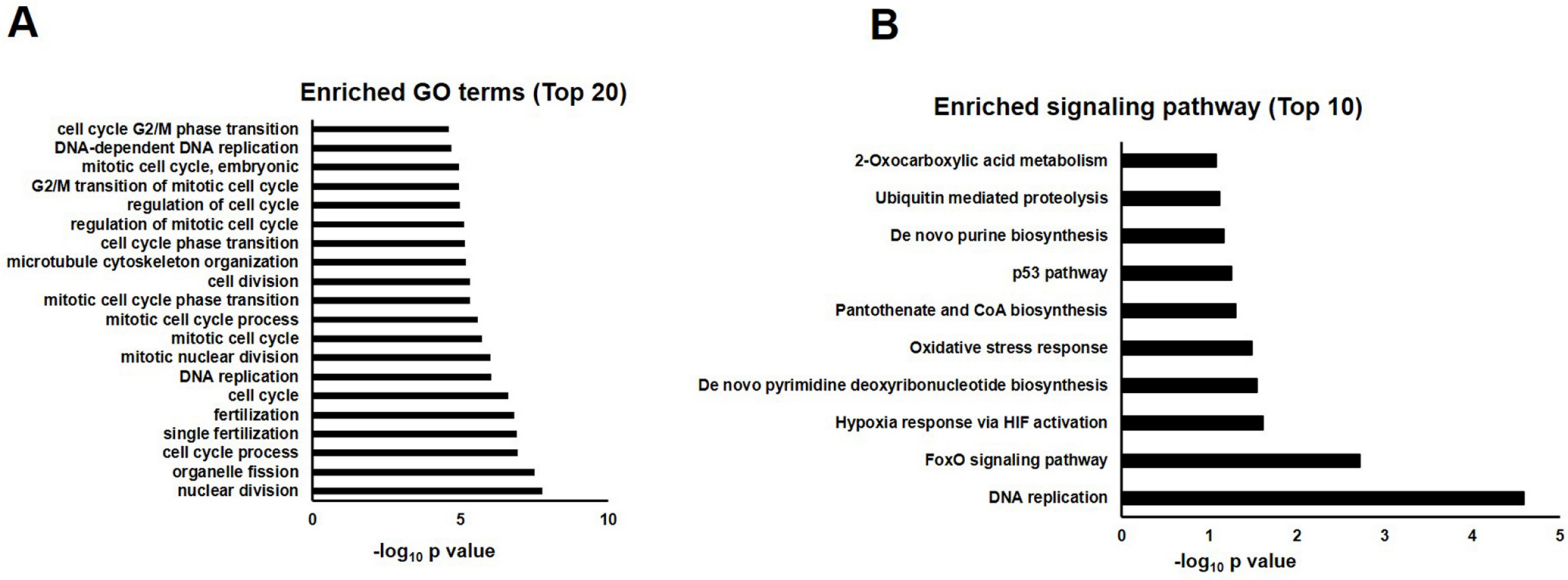
GO annotation and pathway enrichment of differentially expressed genes in AD flies treated with quercetin or control food (AD Quercetin *vs.* AD DMSO) **A.** Functional annotations were performed to analyze differentially expressed genes in biological process, molecular function and cell component in AD *Drosophila* fed with quercetin or control food. Top 20 GO terms were shown and displayed as -log_10_ *P* values. **B.** Differentially expressed genes were analyzed by KEGG pathway, PID Curated, PID BioCarta, PID Reactome, BioCyc, Reactome and Panther. Top 10 enriched pathways were shown and displayed as -log_10_ *P* values.

**Figure 5:**
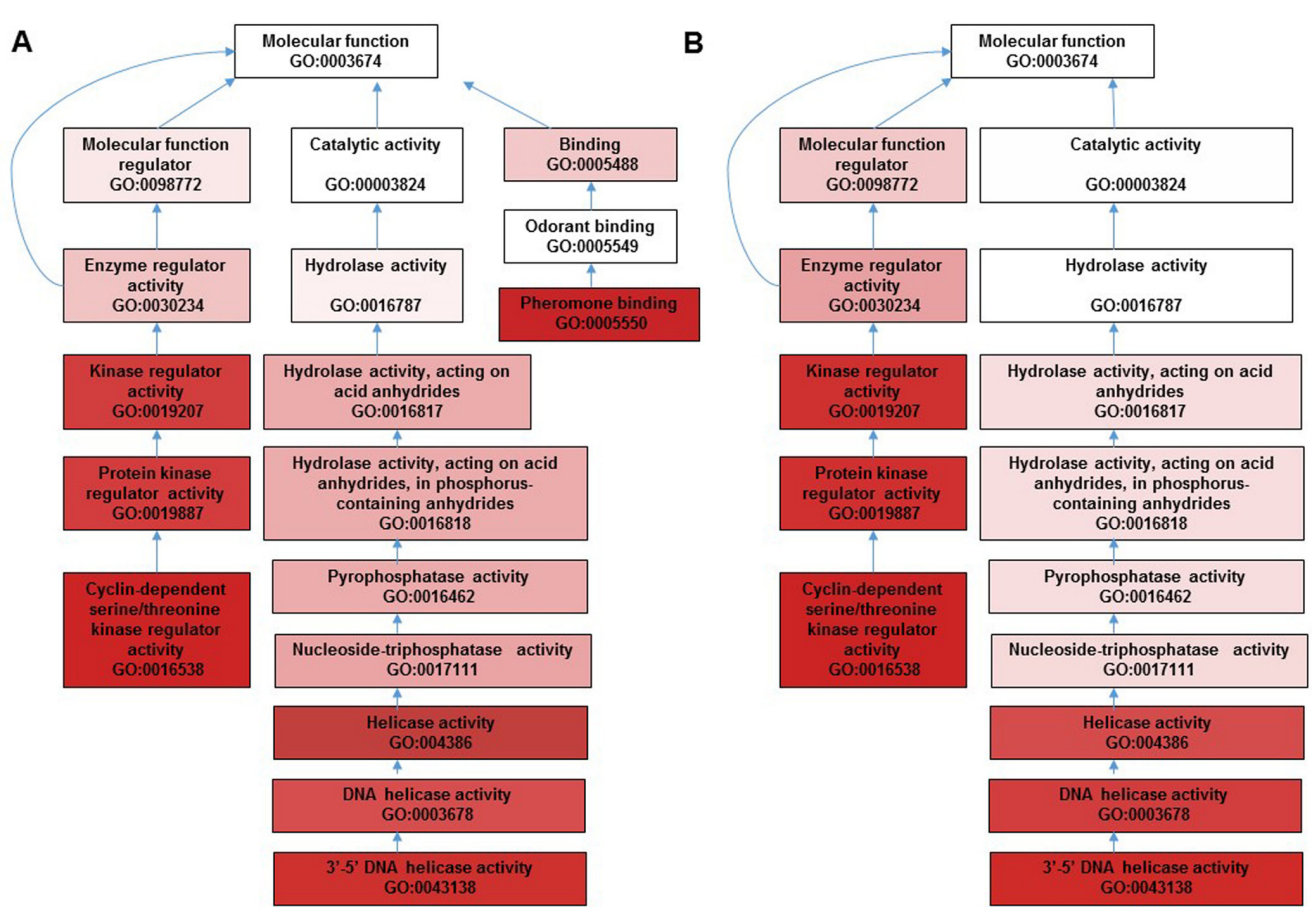
GO enrichment hierarchy for terms interaction in molecular function for dysregulated genes in AD *vs.* WT and AD Quercetin vs. AD DMSO

In order to investigate the mechanism underlying quercetin's beneficial effects on AD *Drosophila*, we compared gene expression profiles of AD flies fed with quercetin (AD Quercetin) or control food (AD DMSO) for 10 days. We found 5 transcripts were upregulated while 74 transcripts were suppressed. Gene ontology analysis showed that a plethora of clusters in biological process, cell component and molecular function which had been interrupted in AD flies were reversed by quercetin (Figure 4A). GO enrichment hierarchy of molecular function demonstrated that, compared with control flies, differentially expressed genes in AD flies were enriched in clusters related to cyclin-dependent protein serine/threonine kinase regulator activity, 3′-5′ DNA helicase activity and pheromone binding (Figure 5A). Interestingly, when we analyzed GO enrichment hierarchy of molecular function for quercetin targeting genes in AD flies, clusters including cyclin-dependent protein serine/threonine kinase regulator activity and 3′-5′ DNA helicase activity were also identified as enriched terms (Figure 5B). Pathway analysis showed that DNA replication and cell cycle proteins in FoxO signaling pathway were the most enriched pathways targeted by quercetin in AD flies (Figure 4B).

**Figure 6:**
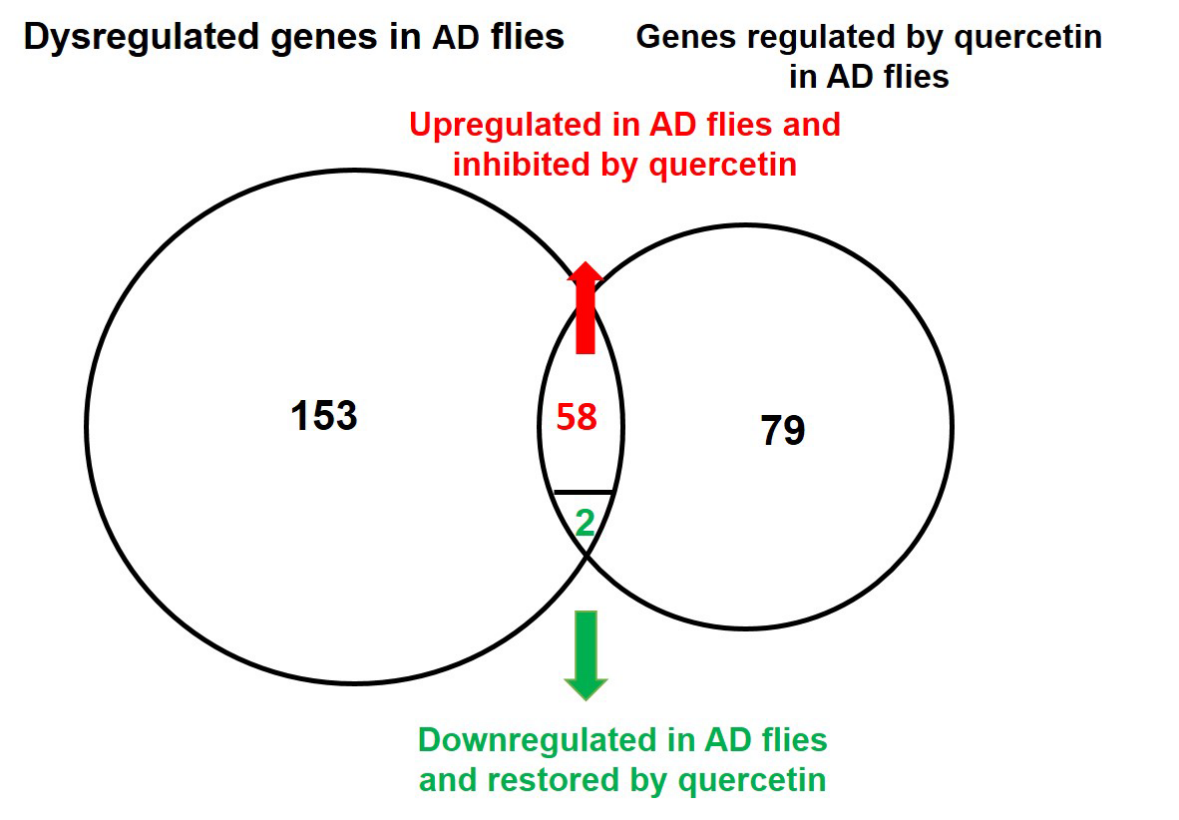
Venn diagram of genes dysregulated in AD flies and rescued by quercetin 153 transcripts were dysregulated in AD flies as compared with control. The expression of 79 transcripts were influenced by quercetin in AD flies. When these results were combined together, it indicated that 58 Aβ induced and 2 Aβ suppressed genes were restored by quercetin.

Comparing the data of AD *vs.* WT and AD Quercetin *vs.* AD DMSO, we found the expression of 59 Aβ upregulated transcripts (58 genes) and 2 Aβ suppressed genes were restored by quercetin feeding (Figure 6). Quercetin restored the perturbation induced by Aβ on genes in 16 pathways, including DNA replication, cell cycle proteins in FoxO signaling pathway, Hypoxia response *via* HIF activation, *De novo* pyrimidine deoxyribonucleotide biosynthesis, Oxidative stress response and p53 pathway. Quercetin targets in these pathways were listed in Table 2. Collectively, these results indicated that quercetin could effectively restore genes related with cell cycle and DNA replication in AD *Drosophila*.

**Table 2:** Dysregulated pathways in AD flies rescued by quercetin treatment

Pathway	genes	Up or down regulated by quercetin in AD flies
DNA replication,	Mcm7, Mcm3, PCNA, Mcm2	down
FoxO signaling pathway	CycB, polo, CycB3	down
Hypoxia response via HIF activation	dhd	down
*De novo* pyrimidine deoxyribonuleotide biosynthesis	RnrL	down
Oxidative stress respose	dhd	down
p53 pathway	CycB	down
*De novo* purine biosynthesis	RnrL	down
Unbiquitin mediated proteolysis	fzy, APC7	down
Mismatch repair	PCNA	down
Base exicision repair	PCNA	down
Doso-vental axis formation	orb	down
Nucleotide excision repair	PCNA	down
Glutathion metabolism	RnrL	down
Pyrimide metabolism	RnrL	down
Purine metabolism	RnrL	down
Metabolic pathways	RnrL, CG1673, CG5966	down

**Figure 7:**
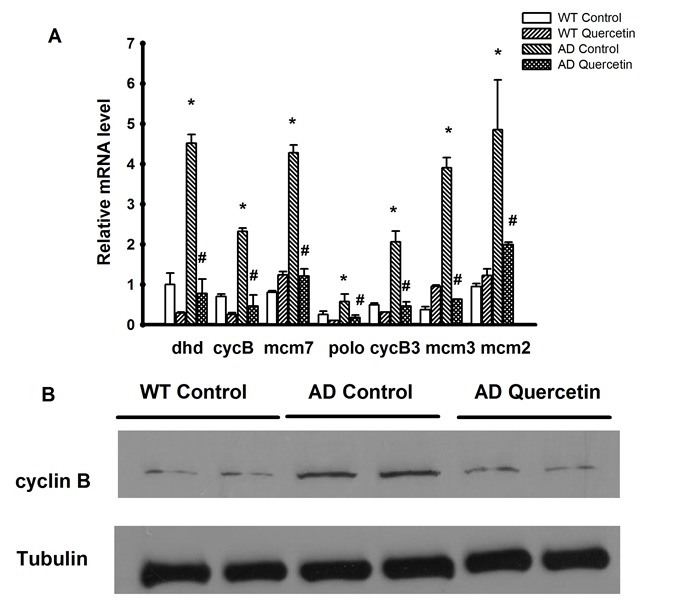
Validation of microarray results by qRT-PCR and Western blot **A.** The mRNA levels of dhd, cyclin B, mcm2, mcm3, mcm7, cyclin B3 and polo were measured in WT and AD flies treated with quercetin or control food. The data was presented as the average±SEM (* *P* < 0.05 between WT Control and AD Control flies. # *P* < 0.05 between AD Control and AD Quercetin flies). **B.** Western immunoblot analysis of cyclin B and α-tubulin using the indicated antibodies. Representative results from three independent experiments are shown.

In order to confirm our findings, we selected 7 genes including cyclin B, cyclin B3, polo, mcm2, mcm3, mcm7 and dhd to validate whether the Aβ induced targets dysregulation could be restored by quercetin. Cyclin B, cyclin B3 and polo are cell cycle proteins in FoxO signaling pathway. Additionally, mcm2, mcm3 and mcm7 are DNA helicases essential for DNA replication. The qPCR results were consistent with microarray data (Figure 7A). As most of the dysregulated *Drosophila* genes do not have commercially available antibodies, we could only validate the protein level of cyclin B by western blot. The cyclin B antibody was obtained from Santa Cruz Biotechnology (sc-15872) and has been proved to be specific and sensitive for western blot [21]. Consistent with mRNA expression, cyclin B protein level was increased in the brain of AD *Drosophila* and restored after feeding with quercetin (Figure 7B).

### Protein-protein interaction network for transcriptomic results

**Figure 8:**
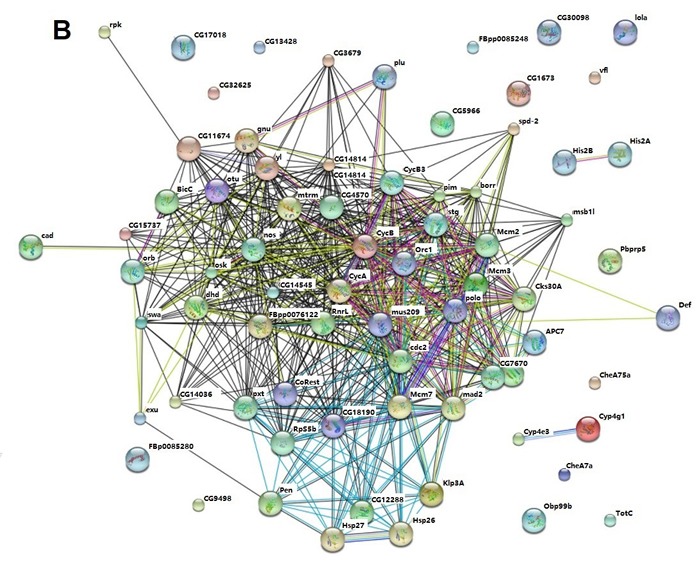
STRING analysis of the relationship between differentially expressed genes **A.** The differentially expressed (DE) genes between AD and WT flies were analyzed using the STRING database. The network nodes represent the proteins encoded by the DE genes. Lines linking nodes with different colored represent types of evidence used in predicting (red line: fusion evidence; green line: neighborhood evidence; blue line: coocurrence evidence; purple line: experimental evidence; yellow line: textmining evidence; light blue line: database evidence; black line; coexpression evidence). **B.** The DE genes between AD Quercetin and AD DMSO were analyzed using the STRING database as described above.

In order to explore the possible protein-protein interaction between dysregulated genes induced by Aβ, STRING (http://string-db.org/), a database of known and predicted protein interactions was used to analyze microarray data [22]. As shown in Figure 8A, cyclin B (cycB), cyclin B3 (cycB3), polo, mcm2, mcm3, mcm7 and mus209 (PCNA) were hubs of the network and interact with other dysregulated proteins through direct or indirect manner. These nodes interacted with each other as shown in the figure. The results indicated that cell cycle related genes served as nodes and collaborated with other dysregulated proteins to form networks and mediate Aβ toxicity in *Drosophila*.

Protein-protein interaction network for quercetin targeted genes in AD *Drosophila* was also analyzed by STRING. Interestingly, protein-protein interaction networks were consistent with that of AD *vs.* WT which identified cycB, cycB3, polo, mcm2, mcm3, mcm7 and mus209 (PCNA) as hubs of the network and collaborating with other proteins (Figure 8B). It indicated that quercetin could ameliorate AD phenotypes by rescuing cell cycle related signaling pathways and protein interaction networks.

### Inhibition of cell cycle protein cyclin B could ameliorate AD phenotypes

**Figure 9:**
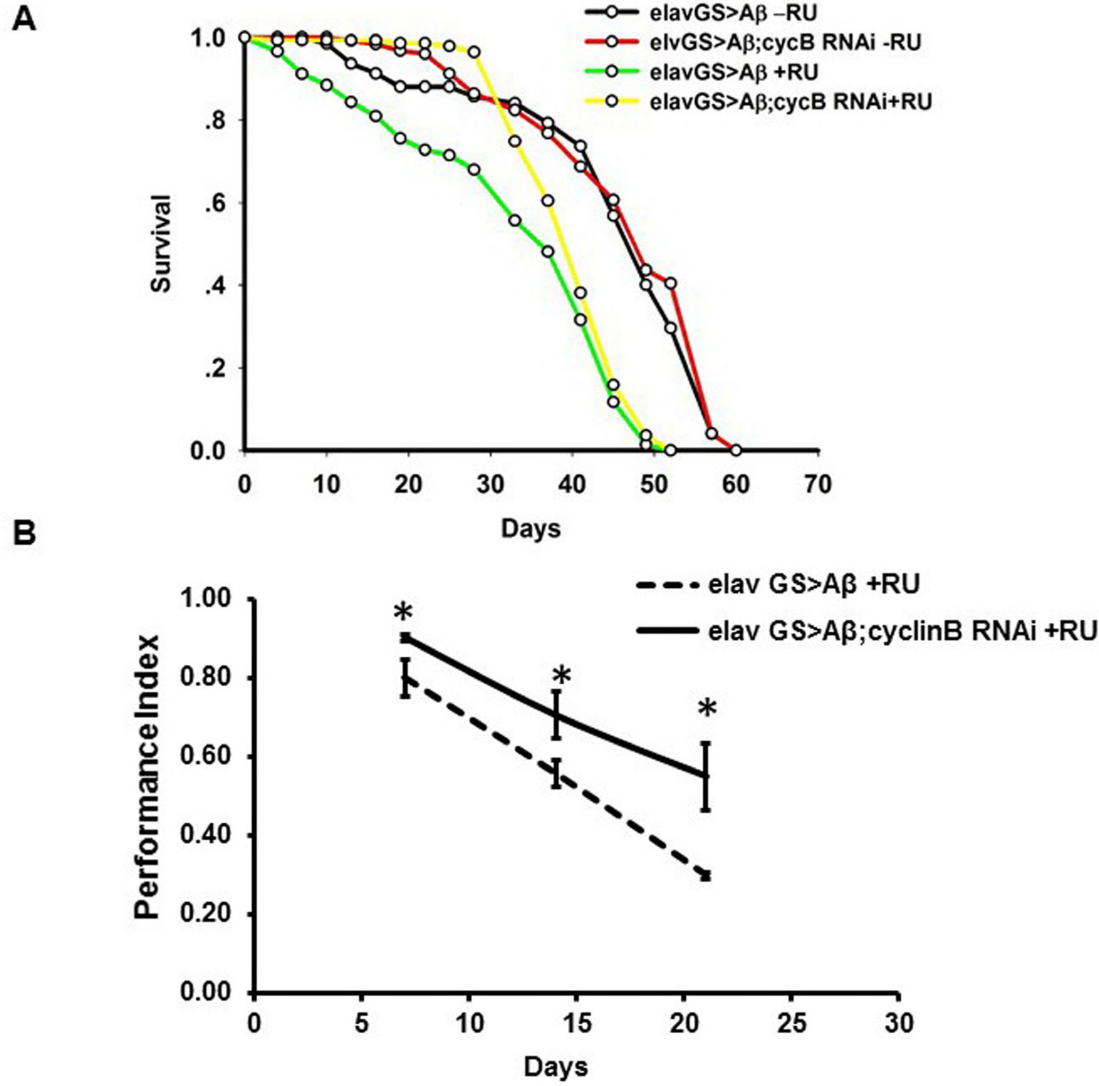
*In vivo* RNAi of cyclin B ameliorated AD phenotypes in *Drosophila* **A.** The experiments were performed independently for 3 times and representative results were shown. Survival curves were compared using the log-rank test and significant difference was observed between RU induced and uninduced groups (*P* = 4.62276×10^-18^ between elavGS > Aβ-RU and elavGS > Aβ+RU). Additionally, cyclin B RNAi extended the lifespan of AD fruit flies (*P* = 0.000611737 between elavGS > Aβ+RU and elavGS > Aβ;cyclin B RNAi +RU). **B.** Climbing abilities were presented as the average performance index (PI)±SEM (* *P* < 0.05 between between elavGS > Aβ+RU and elavGS > Aβ;cyclin B RNAi +RU).

As an important cell cycle protein and hub of protein-protein interaction networks mentioned above, cyclin B was chosen to further validate our hypothesis. In order to verify our conclusion, cyclin B RNAi in the brain were induced by RU486 in adult-onset AD *Drosophila*. We crossed *elav*-GeneSwitch (ElavGS) line with flies carrying cyclin B siRNA and Arctic Aβ_42_. The offspring will not express siRNA and Aβ in absence of Mifepristone (RU486, RU). We transferred these flies to RU foods after eclosion and cyclin B siRNA expression would be induced in pan-neuronal manner together with Aβ, which could rule out their effects on development. We found that knocking down cyclin B expression could effectively extend the lifespan and improve locomotive defects (Figure 9). Taken together, our results indicated that quercetin could ameliorate AD pathogenesis by targeting cell cycle related pathways perturbed by Aβ overexpression.

## DISCUSSION

Quercetin has been reported to inhibit Aβ toxicity *in vitro* and *in vivo*. However, the detailed mechanisms are still elusive. Using *Drosophila* models, we found quercetin could extend lifespan and rescue climbing ability of AD flies, suggesting its potential therapeutic application in AD. Transcriptomic profiling and functional annotation analysis showed that cell cycle related proteins were interrupted by Aβ. Gene ontology analysis showed these genes were enriched in terms related to cell cycle and DNA replication. These terms interact with each other to form hierarchies. Further analysis using STRING database showed that proteins in these pathways formed a network which contributed to AD pathogenesis. Dietary supplementation of quercetin could rescue these cell cycle related signaling pathways to ameliorate AD phenotypes.

The mechanisms for the beneficial effects of quercetin in AD could be explained in several aspects. By inhibition of BACE1-mediated cleavage of APP, quercetin suppresses β-amyloid synthesis in cell-free, cell-based and in silico studies [23].Using primary Tg2576 AD mouse neurons, quercetin metabolite quercetin-3-O-glucuronide has been proved to significantly reduce the generation of β-amyloid [24]. In addition to Aβ synthesis, quercetin inhibits formation and extension of Aβ fibrils and stimulates destabilization of preformed Aβ fibrils [25, 26]. Quercetin-3-O-glucuronide has also been identified to interfere with the initial protein-protein interaction of Aβ_40_ and Aβ_42_ which is necessary for Aβ oligomerization [24]. Oxidative stress is usually found in AD cases and believed to contribute to disease progression. As a potent antioxidant, quercetin is able to effectively clear reactive oxygen species which could be beneficial to AD [27, 28]. Quercetin and its metabolites have been reported to act on several signaling pathways, including cAMP-response element binding protein (CREB), c-Jun N-terminal kinases, the mitogen-activated protein, macroautophagy, calcium homeostasis, proteasomal degradation and GADD34-eIF2α-ATF4 pathways that participate in AD pathogenesis [29-32]. Moreover, quercetin serves as Sirt1 agonist and acetylcholine-esterase (AchE) inhibitor to ameliorate AD phenotypes [33, 34]. However, there are still some controversial reports. Dietary supplementation of quercetin (2 mg/g diet) for 6 weeks has no effects on expression and activities of α and β secretase in mice cortex [35]. The expression of neprilysin, heme oxygenase-1 and γ-glutamylcysteine synthetase are not changed. Further investigation with transcriptomic analysis is required to clarify the detailed mechanisms.

Using microarray analysis for wild type and AD flies fed with quercetin or control food, we elucidated that quercetin ameliorated AD phenotypes in *Drosophila* by targeting several signaling pathways, such as DNA replication, FoxO signaling and Hypoxia response *via* HIF activation. Cell cycle proteins in FoxO signaling such as cyclin B, cyclin B3 and polo as well as DNA replication proteins including mcm2, mcm3, mcm7 and PCNA were the hubs of protein-protein interaction network. Their expression was dysregulated by Aβ expression and rescued by quercetin. Our results support the well-recognized theory that ectopic cell cycle events could mediate neurodegeneration in AD [36]. In AD brains, neurons expressing biomarkers of cell cycle progression and DNA replication are vulnerable to Aβ toxicity. Cyclin B is only expressed in the severely affected brain regions of AD patients such as subiculum, dentate gyrus, CA1 region hippocampus, ‘locus coeruleus, and dorsal raphe nuclei [37-39]. The expression of Swedish double mutant APP (Swe-APP) in differentiated PC12 cells, rat primary cortical neurons and Tg2576 mice brain tissues increases mRNA and protein level of cyclin B1 [40]. Normally, neurons are arrested at an early stage of the cell cycle and able to re-differentiate. However, cell cycle is allowed to progress into the G2 phase in Alzheimer's disease. G1/S checkpoint has been bypassed and that the cell cycle is arrested in G2 when cyclin B is expressed in neurons. Neurons arrested at the G2/M phase of the cell cycle are susceptible to AD before they die [41, 42]. neurons which are not able to undergo re-differentiation will die *via* an apoptotic pathway or produce AD pathology such as tau hyperphosphorylation and Aβ deposition that ultimately leads to neuronal cell death [43].In addition to cyclin B, the expression of PLKs (*Drosophila* polo homologues) is upregulated in susceptible hippocampal and cortical neurons of AD patients [44, 45]. A significant association between the genotypes of PLK2 (rs15009 and rs702723) was found in AD [46]. Mechanism study shows PLK1 is elevated during the cell-cycle re-entry of neuronal cells *in vitro* [47]. Furthermore, inhibition of PLK1 kinase activity or depletion of PLK1 by RNAi reduces Aβ induced neuronal cell death. These results proposed that cell cycle proteins such as cyclin B, cylin B3 and PLKs as possible targets for AD treatment. Consistent with these reports, we found cyclin B expression was increased in AD flies and restored by feeding quercetin both at mRNA and protein level. Additionally, inhibition of cyclin B expression by RNAi could ameliorate AD phenotypes. We proposed that neuroprotective effects of quercetin in AD were mediated at least partially by targeting cell cycle related pathways. Supporting our findings, in tau protein induced AD *Drosophila*, ectopic cell-cycle activation mediated by cyclin B and cyclin B3 leads to apoptosis of postmitotic neurons *in vivo* [48]. Additionally, quercetin has been found to suppress cyclin B expression in liver, breast carcinoma and human leukemic T-cells, which could induce cell cycle arrest, decrease cell proliferation and enhance apoptosis [49-51].

DNA replication is a subsequent cascade for cell cycle re-entry and precedes neuronal cell death in Alzheimer's disease [52, 53]. DNA replication gives rise to neurons with a content of DNA above the diploid level. Neurons with an aneuploid set of chromosomes are rare in the normal brain. In contrast, the number of aneuploid neurons is highly increased in AD [54]. Using fluorescent *in situ* hybridization, four separate genetic loci on three different chromosomes are found to have replicated in hippocampal pyramidal and basal forebrain neurons in AD [55]. In our study, we identified DNA replication pathway proteins including mcm2, mcm3, mcm7 and mus209 (PCNA) were upregulated in AD flies and suppressed after quercetin dietary supplementation. Among them, mcm2, mcm3, mcm7 are DNA helicases and PCNA acts as a scaffold to recruit proteins involved in DNA replication. Consistent with our findings, quercetin was reported to inhibit DNA replication in human HL-60 leukemic cells [56]. Lin HH found quercetin could inhibit DNA helicase *in vitro* [57]. As an important replication factor, Mcm2 phosphorylation is associated with AD pathology, such as neurofibrillary tangles, neuropil threads, and dystrophic neurites [58]. Mcm2, Ki67 and PCNA expression in neurons and glial cells increases significantly and associates with higher burdens of Alzheimer-type pathology. Taken these findings and our results together, cell cycle disturbances in AD and related DNA replication may contribute to AD pathology and explain the mechanism of quercetin's therapeutic effects.

Aβ induced dysregulation of genes involved in cell cycle events are mediated by activation of signaling pathways such as GSK-3β and ERK1/2 in AD [59, 60]. Interestingly, it has been reported that quercetin could inhibit GSK-3β and ERK1/2 pathways in the brain [61, 62]. In control flies, these pathways were not activated and quercetin's effects on the expression of cell cycle related genes were not as significant as in AD *Drosophila*.

Our study revealed that quercetin could ameliorate AD pathogenesis in *Drosophila* model mediated through modulating the expression of cell cycle related proteins such as cyclin B. It broadened the understanding about the mechanism of quercetin's beneficial effects on AD and provided new insights into the therapeutic methods for neurodegeneration.

## MATERIALS AND METHODS

### Fly stocks and maintenance

Flies were maintained at 25°C on 12h:12h light: dark cycle. UAS- ArcAβ_42_ flies were from Dr. D. Crowther (University of Cambridge, UK). The *elav*-Gal4^c155^ stocks were from Bloomington *Drosophila* Stock Center. Cyclin B RNAi line was obtained from Dr. Norbert Perrimon, at Harvard Medical School, and Dr. Jian-Quan Ni at Tsinghua Fly Center. ElavGS line was originally made by Prof. H Keshishian (Yale, USA). Flies used in all experiments were backcrossed six times into the *w^1118^* genetic background. Quercetin was obtained from Jianfeng Health Tech. Co., Ltd (Zhejiang, China). HPLC, Mass spectrometry and NMR was performed to quantify quercetin purified from flowers of *Styphnolobium japonicum*. Quercetin was dissolved in DMSO and then added to standard yeast/agar (SYA) food at final concentration of 0.44g/L (1.31mM) which was much lower than those used in mouse model [63]. For control food, DMSO alone was added.

### Lifespan analyses

Lifespan assay was performed as described previously [62]. Mated females were discriminated and transferred into vials with standard sugar-yeast medium supplemented with quercetin or equivalent concentration of DMSO at the density for 10 flies in each vial. Vials were changed 3 times a week and at least 100 in total were analyzed for survival in each group. For lifespan experiments, data are presented as survival curves and analysis was performed using log-rank tests to compare between groups. It is assumed to be significant difference if *P* < 0.05.

### Climbing assay

25ml plastic pipettes were used to analyze *Drosophila* climbing ability. Each group contains 3 vials and twenty female flies were cultured in each vial. Flies transferred into an empty pipette. After gently tapped to the bottom, the numbers of flies that could climb to the top (above the 25ml line) and stayed at bottom (below the 2ml line) within 45 seconds were recorded. The climbing was analyzed 3 times for every vial at each time point. The performance index (PI) was calculated as described previously [63].

### RNA isolation

50 fly heads were harvested and homogenized in Trizol reagent (ambion). After centrifugation at 12,000g for 10min at 4°C, the supernatant was transferred to a new tube and then mixed thoroughly with chloroform. After incubation and centrifugation at 12,000g for 15min at 4°C, aqueous phase was transferred into new tubes. RNA was precipitated by isoproponal and washed with 75% ethanol. Finally, RNA pellets were dissolved in RNase free water. RNA concentration and OD260/280 ratio were measured by ultraviolet spectrophotometer.

### Affymetrix *Drosophila Genome* 2.0 array

Transcriptomic analysis was performed by Affymetrix *Drosophila* Genome 2.0 Array according to the manufacturer's instructions. Briefly, reverse transcriptase was used to synthesize fist-strand cDNA using oligo dT. After RNA digestion in DNA-RNA hybrid, second strand cDNA was produced accordingly. *In vitro* transcription was performed with T7 Enzyme to make biotin labeled cRNA (Ambion #1792 cRNA kit), which was further purified by RNA Binding Beads. After fragmentation, cRNA was hybridized with probes in chips, followed by washing, staining and scanning. Affymetrix GeneChip Command Console Software was used to abstract and analyzed the data. SAM (significance analysis of microarray) of R software and RMA method were used to identify differentially expressed genes (Ratio≥2 or Ratio≤0.5) as published literature [64].

### Gene ontology and pathway enrichment analysis

KOBAS (KEGG Orthology Based Annotation System) was used to analyze gene ontology and pathway enrichment for dysregulated genes. Gene ontology was analyzed by the databases and divided into three categories: biological process, molecular function and cell component. *P* values were calculated according to published literature [65]. In order to show the interaction for different terms, GO hierarchy for significant influenced terms (*P* < 0.05) in biological process, molecular function and cell component were drawn. Pathway enrichment were analyzed by KEGG pathway, PID curated, PID BioCarta, PID reactome, BioCyc, Reactome and Panther.

### Protein interactions networks assay

STRING is a database of known and predicted protein interactions.The interactions include direct (physical) and indirect (functional) associations; they are derived from different sources including Genomic Context, High-throughput Experiments, Coexpression and Previous Knowledge. The nodes of network represent the proteins encoded by the dysregulated genes. Different colored lines link of nodes represent types of evidence used in predicting associations. (red line: fusion; green line: neighborhood; blue line: concurrence; purple line: experimental evidence; yellow line : textmining evidence; light blue line: database evidence; black line: coexpression evidence).

### Quantitative real-time PCR analysis

Quantitative real-time PCR (qRT-PCR) analysis was performed to validate the data obtained from microarray. Fist strand cDNA was sysnthesized using PrimeScript™ RT reagent Kit with gDNA Eraser (RR047A Takara) according manufacturer's instructions. M-MLV reverse transcriptase (Takara). 1μg total RNA was mixed with 2μl gDNA Eraser Buffer, 1μl gDNA EraserRT and RNase free water to a 10μl final volume and incubated at 42°C for 5 min. The mixture was further supplemented with 1μl PrimeScript RT Enzyme Mix I, 1μl RT Primer Mix, 4μl PrimeScript Buffer 2 and 4μl RNase free water and then incubate at 37°C for 15 min and followed by 85°C for 5 sec. qPCR reaction was performed with 2×SYBR Green PCR Master Mix (Takara) and ABI StepOnePlusTM real-time PCR System (Applied Biosystems) with the program: 95°C for 5 min to denature DNA templates, followed by 40 cycles of 95°C for 15 s, 60°C for 30s, and 72°C for 32 s. The PCR primers were listed in Table 3.

**Table 3:** Primers for qRT-PCR validation experiments

Gene	Primer sequence
mcm7	Forward: 5′-CATCATGACGACCCTAAACG-3′
	Reverse: 5′-ACAGCAGATCGAAACGTGAG-3′
mcm3	Forward: 5′-TAATCCGGTGTACGGAAGGT-3′
	Reverse: 5′-GTCCAGCATAACGAAGAGCA-3′
mcm2	Forward: 5′-GACGCACGTAATGGACAATC-3′
	Reverse: 5′-CTGATCTCCCAGGATCTCGT-3′
cyclin B	Forward: 5′-CAGTTCTTCCGAGAACGTGA-3′
	Reverse: 5′-TTCCTTCTTGGTCTCTGCCT-3′
polo	Forward: 5′-ATGGAGCTGCACAAACGTAG-3′
	Reverse: 5′-ATAATGCGGTTATCGTGCAA-3′
cyclin B3	Forward: 5′-TTGCTGACACTCCCAGAGAC-3′
	Reverse: 5′-GCGCACTTTGAGGTAGTTGA-3′
deadhead	Forward: 5′-TTTAGCTTGTAAGCGCGAGA-3′
	Reverse: 5′-GCGCCACAAATCATAGCATA-3′
Actin	Forward: 5′-AGAAGGACTCGTACGTGGGT-3′
	Forward: 5′-CATATCGTCCCAGTTGGTCA-3′

### Western analysis

Western blotting was performed as described previously [66]. 20 fly heads were homogenized and underwent SDS-PAGE before transferred to PVDF membranes (Immobilon-P, Millipore). The membrane was blocked with 5% milk and incubated with anti-cyclin B antibody (Santa Cruz Biotechnology sc-15872 which has been proved to be specific and sensitive for western blot [21]), or anti-tubulin monoclonal antibody (1:5000, Sigma) overnight at 4°C. After incubation with HRP conjugated donkey anti-goat or goat-anti-mouse secondary antibody and reacted with SuperSignal West Pico chemiluminescent substrate (Pierce), tubulin and cyclin B expression measured by exposure to X-Omat BT film (Eastman Kodak)

## SUPPLEMENTARY MATERIALS FIGURES


