# Supplementary material for: Quercetin ameliorates Aβ toxicity in Drosophila AD model by modulating cell cycle-related protein expression
Source: Oncotarget. 2016 Sep 10;7(42):67716–31. doi: 10.18632/oncotarget.11963 (PMC5356514; doi:10.18632/oncotarget.11963)
Supplement: Supplementary file 1 [file oncotarget-07-67716-s001.pdf]

Quercetin ameliorates Aβ toxicity in *Drosophila* AD model by modulating cell cycle-related protein expression

Supplementary Material

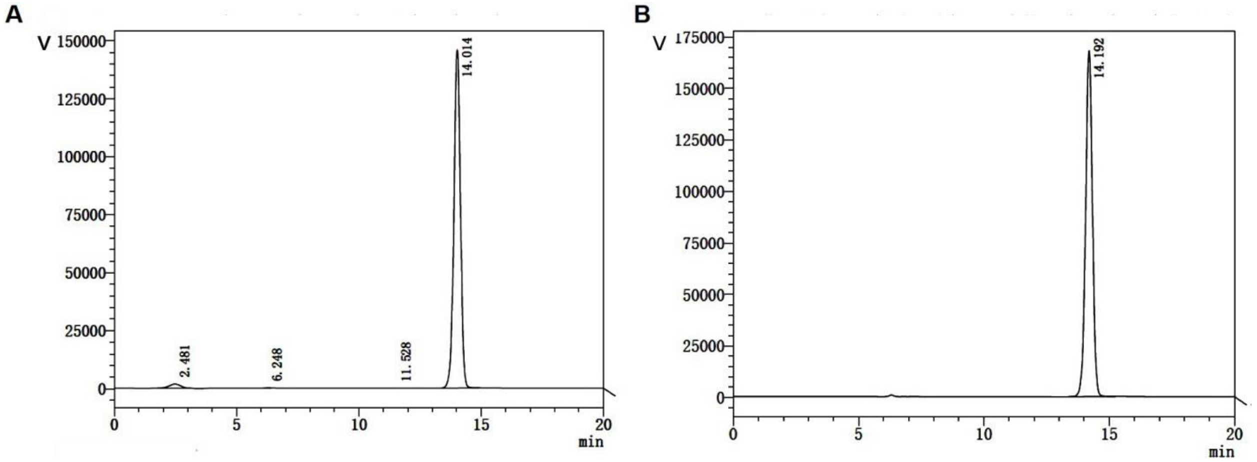

Figure. S1: HPLC chromatogram of quercetin. A: samples extracted from flowers of *Styphnolobium japonicum*. B: standard quercetin.

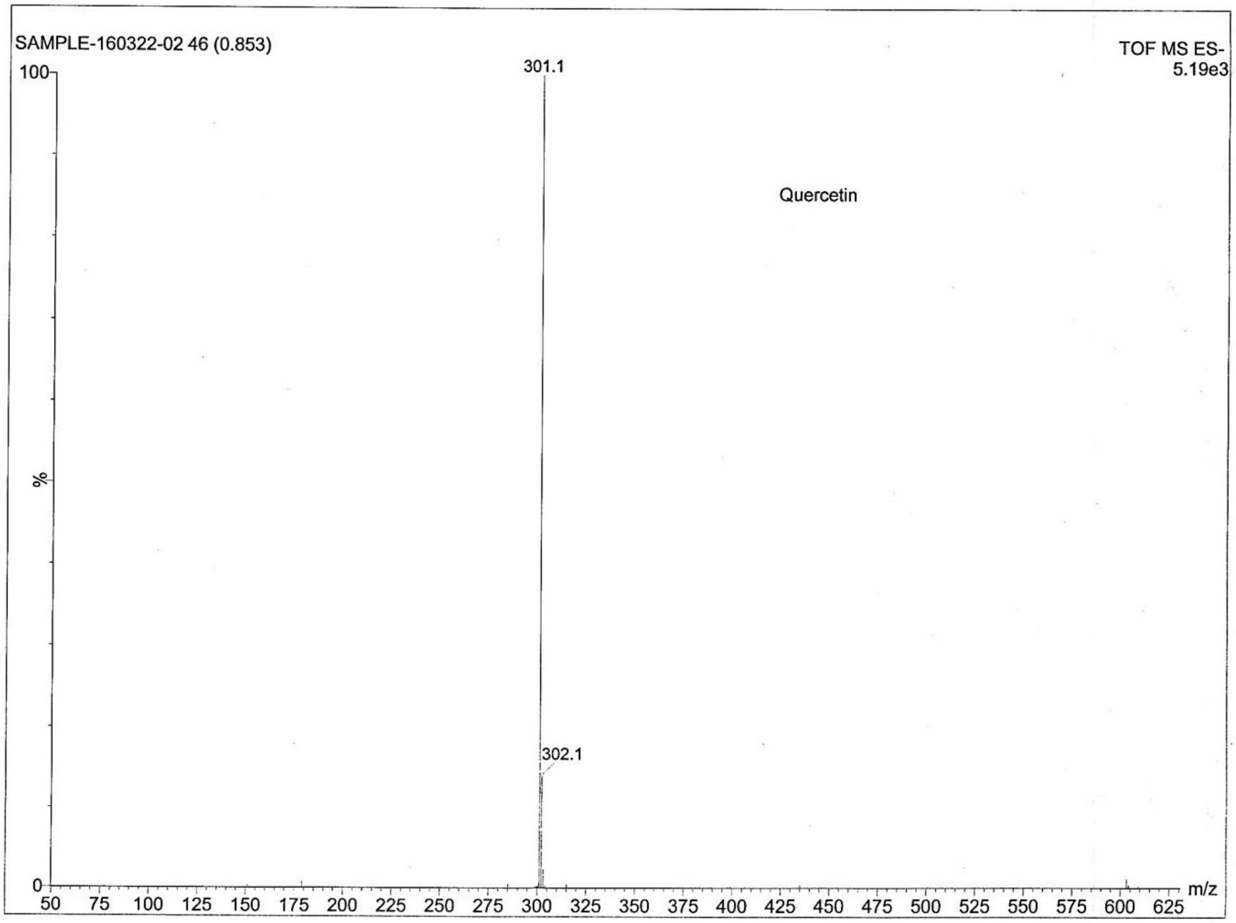

Figure S2: TOF MS analysis of quercetin used in this study.

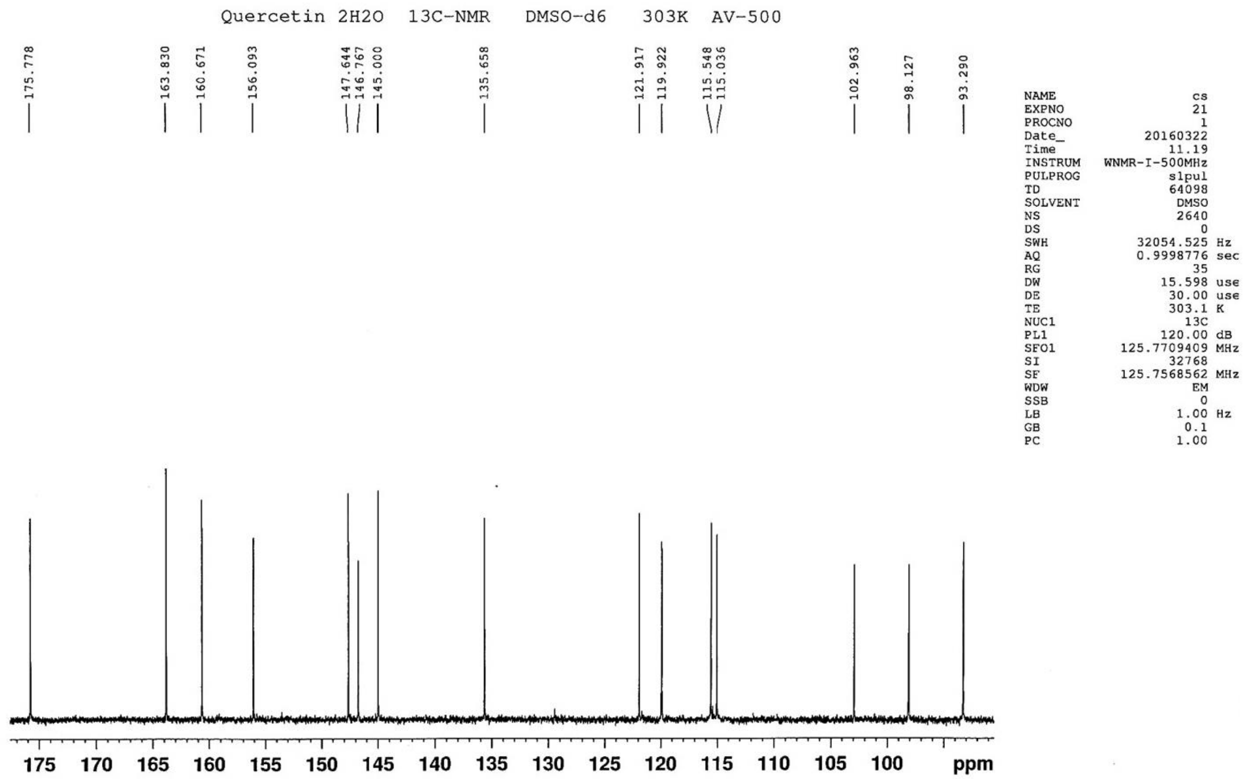

Figure S3: ^{13}C-NMR analysis of quercetin used in this study.

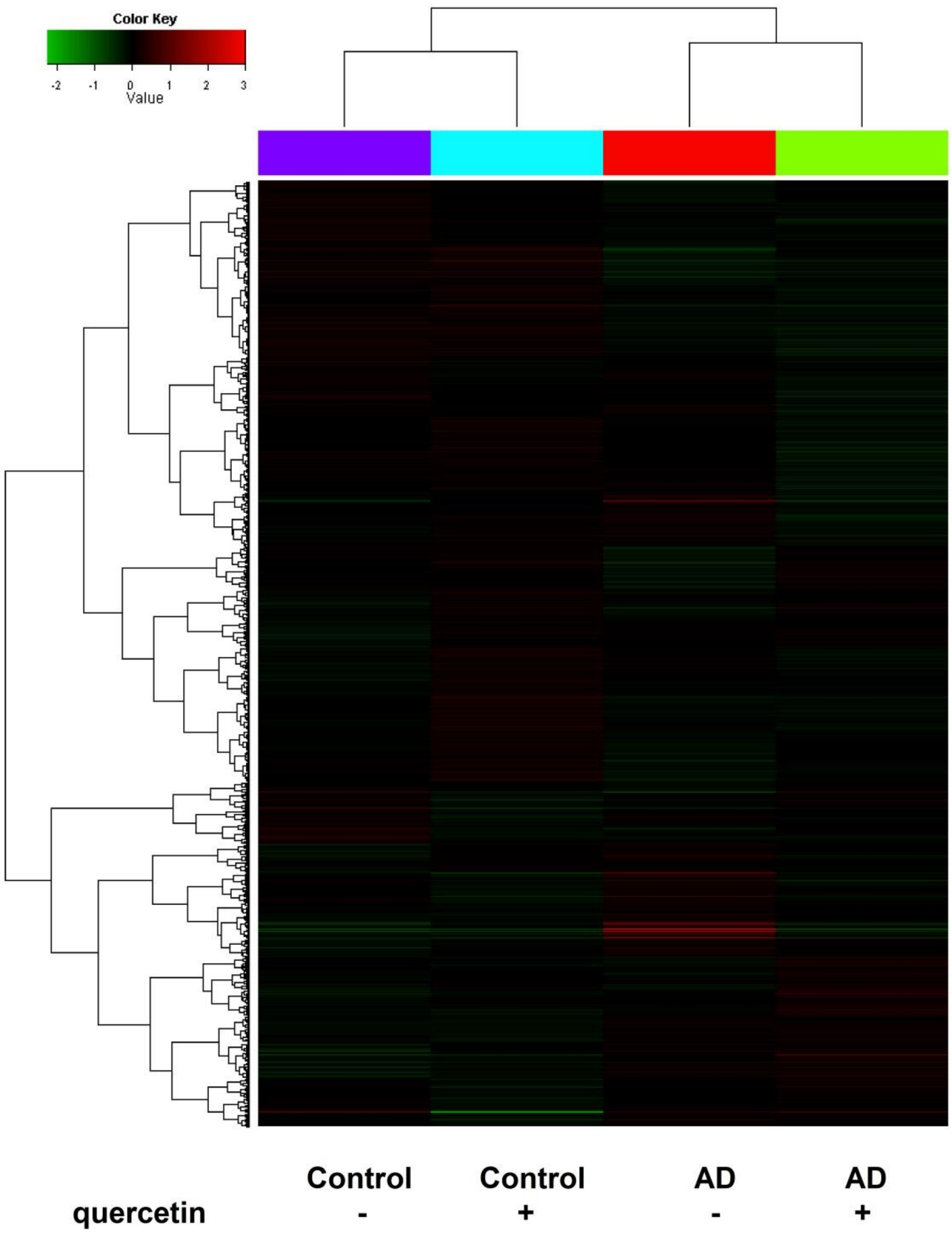

Figure S4: Heatmaps of gene expression following quercetin or DMSO treatment in WT and AD flies at Day 10. Transcriptomic analysis was performed by Affymetrix *Drosophila* Genome 2.0 Array and further analyzed by Cluster 3.0 and Treeview.
